# Uterine carcinosarcoma with heterologous osseous elements: a case report of an extremely rare clinical occurrence with literature review

**DOI:** 10.3389/fonc.2025.1505504

**Published:** 2025-02-14

**Authors:** Hiba A. Al Dallal, Taylor H. Jacobs, Cody L. Bergman, Siddharth Narayanan, Arshi Kaur, Samer Z. Al-Quran, Harpreet Kaur Chopra

**Affiliations:** ^1^ Department of Pathology and Laboratory Medicine, University of Louisville, Louisville, KY, United States; ^2^ Nationwide Children’s Hospital, Columbus, OH, United States; ^3^ Department of Surgery, Virginia Common Wealth University, Richmond, VA, United States

**Keywords:** uterus, tumor, carcinosarcoma, osteosarcoma, cancer, malignant

## Abstract

Carcinosarcoma is a rare and aggressive malignant neoplasm that predominantly affects elderly postmenopausal women and can involve various gynecologic organs. It is characterized by the presence of both malignant epithelial and sarcomatous components. While most uterine carcinosarcomas (UCS) are homologous (sarcomatous component consisting of elements native to the uterus), heterologous components are less common and may vary in composition. Rare heterologous elements can include lipomatous (liposarcoma) or osseous elements (osteosarcoma). We present an unusual case of a 31-year-old female with UCS exhibiting osseous heterologous elements. The patient underwent successful surgical resection and remains in remission during follow-up. This case is notable for its rarity, as highlighted by the uncommon age of the patient and the presence of rare heterologous elements in the UCS. Furthermore, it provides new insights into the diverse presentations of UCS and underscores the importance of comprehensive evaluation in understanding its clinical manifestations.

## Introduction

Uterine carcinosarcoma (UCS) is a rare and aggressive malignancy with both epithelial and sarcomatous components. With an annual incidence of 1.7 per 100,000, these tumors make up only 4.1% of uterine malignancies in the United States (1). While uncommon, the incidence of these tumors has been consistently increasing over the past decade (2). These tumors are notoriously difficult to treat due to their high-grade histology at presentation and propensity to metastasize, portending a poor prognosis. According to a study using the Surveillance Epidemiology and End Result (SEER) database, the 5-year overall survival of UCS in the United States is 14%, which pales in comparison to the 62% 5-year survival in endometrial adenocarcinoma (1), while others estimate a 5-year survival rate ranging from 33-39% (3, 4).

In addition, the biphasic nature of the cancer allows the tumor to manifest in various ways that are characteristic to both cell types, further enhancing its malignant potential. The epithelial, or carcinomatous, component, tends to manifest with characteristics of endometrial, serous, or clear cell adenocarcinoma (4). The sarcomatous component, on the other hand, adopts characteristics of mesenchymal tissue. These may comprise a malignant growth of mesenchymal tissue native to the uterus, e.g. leiomyosarcoma, known as a homologous sarcoma or can develop mesenchymal tissue of a different anatomical origin, known as a heterologous sarcoma (3).

Heterologous sarcomatous elements are exceedingly rare, and the most common tissues of this nature include cartilage (chondrosarcoma) or skeletal muscle (rhabdomyosarcoma) while the least common include bone (osteosarcoma) and adipose tissue (liposarcoma) (4). We present an extremely rare case of a 31-year-old female with UCS containing malignant osseous heterologous elements, characteristic of osteosarcoma. She underwent a successful surgical resection and after completing two (out of four) rounds of adjuvant chemotherapy and brachytherapy, shows no evidence of disease during follow-up.

## Case report

A 31-year-old nulliparous Caucasian female was referred for the management of cervical dysplasia following a Pap smear test result indicating ASC-H (atypical squamous cells, cannot exclude high-grade squamous intraepithelial lesion). Her past medical history was significant for obesity and psoriatic arthritis treated with methotrexate. She occasionally consumed alcohol and, in the past, had smoked recreational drugs (marijuana/hashish) but denied history of tobacco use. There was no family history of malignancy, and the patient had never used any hormonal contraceptive methods.

**Figure 1:**
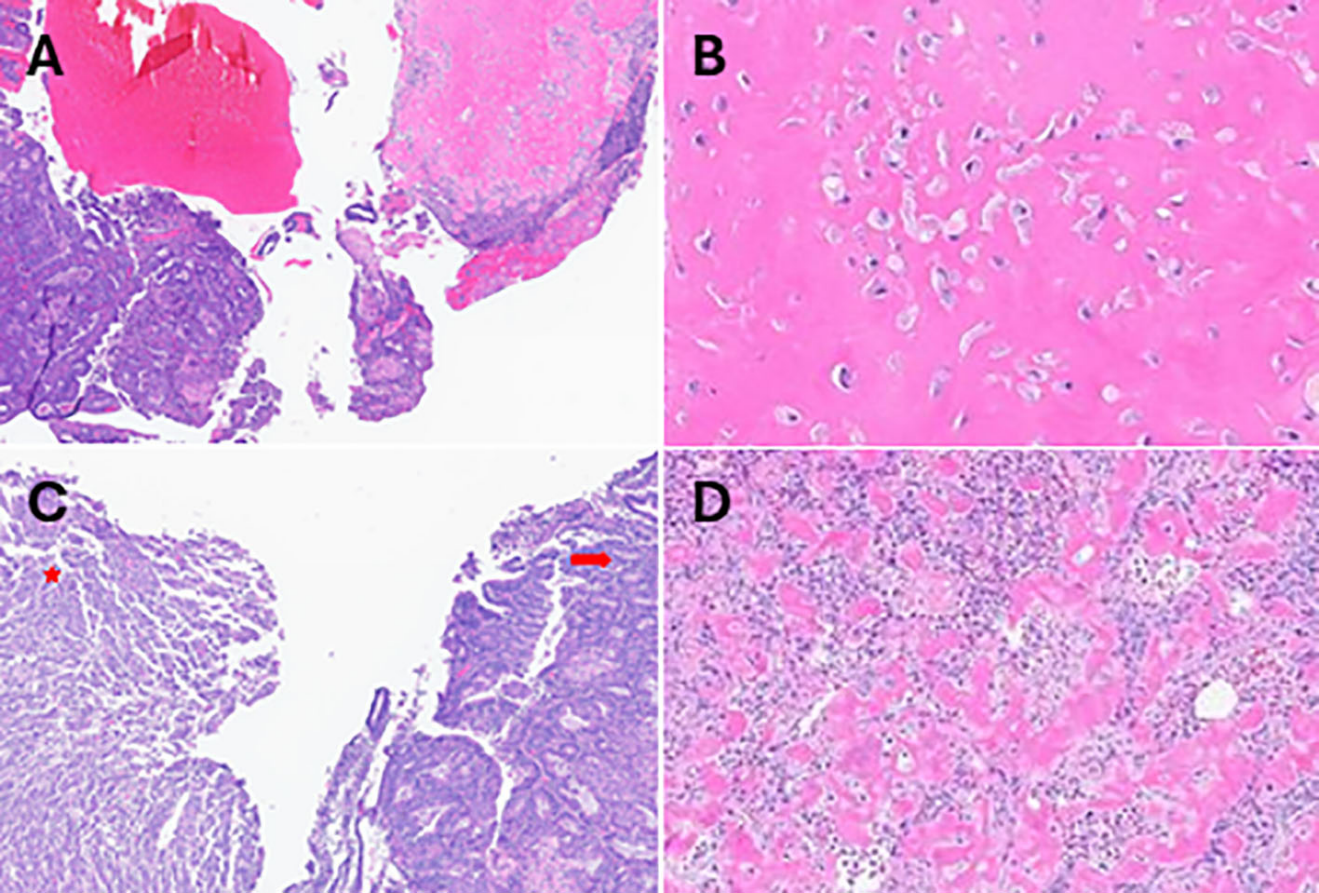
Histopathological evaluations H&E-stained sections obtained after D&C showing **(A)** the biphasic tumor (high-grade carcinoma and osteosarcoma). Densely stained eosinophilic regions indicating **(B)** the osteosarcoma components at 40x magnifications. H&E-stained sections of tissue obtained after hysterectomy revealing regions of **(C)** endometrioid carcinoma (arrow) and sarcoma (star), **(D)** the osteosarcoma component at 20x magnification.

Although a subsequent cervical biopsy was negative for dysplasia, the patient underwent cold knife conization (CKC) and dilation and curettage (D&C) as her complaints at that time included irregular periods as well as variably severe bleeding with intercourse. Microscopic examination of the cervix revealed a focal low-grade squamous intraepithelial lesion and examination of the D&C tissue revealed a malignant tumor consistent with high-grade endometrioid carcinoma with heterologous osteosarcoma elements, consistent of carcinosarcoma arising from an endometrial polyp (**Figures 1A, B**).

Subsequently, she underwent a total robotic hysterectomy, bilateral salpingoophorectomy, and pelvic sentinel lymph node dissection. Gross examination identified a 3.3 x 2.0 x 1.5 cm polypoid mass in the endometrial uterine cavity. Histological evaluation revealed a biphasic tumor characterized by a noninvasive carcinosarcoma composed of 60% high-grade endometrial endometrioid carcinoma and the remaining 40% having a spindled component with osseous heterologous elements consistent with osteosarcoma (**Figures 1C, D**). Adenomyosis was present but not involved by the tumor, and there was no evidence of lymphovascular invasion. All examined lymph nodes were negative for tumor.

**Figure 2:**
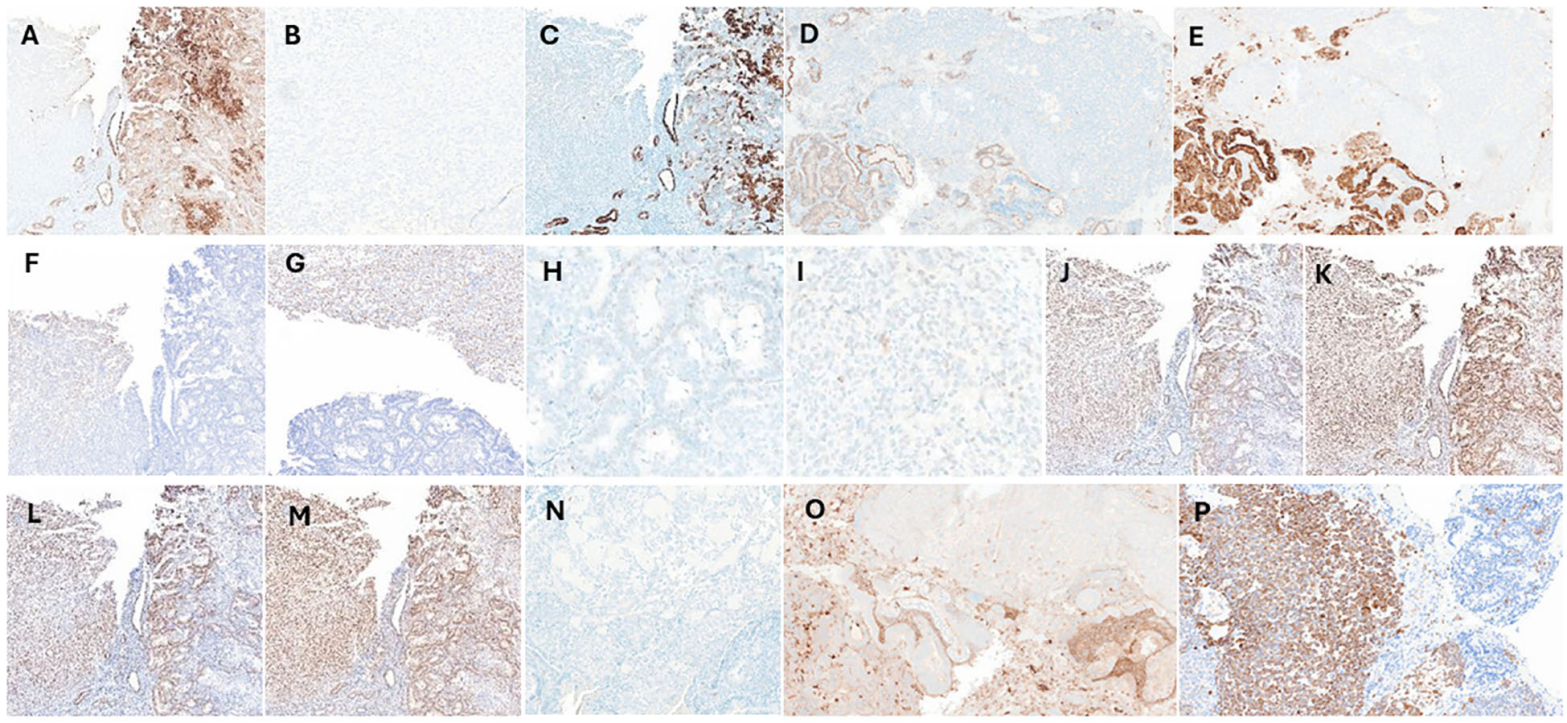
Immunohistochemical (IHC) analysis IHC staining was performed to assess the carcinoma and sarcoma components using various markers including **(A)** the pancytokeratin (CKAE1/3) which stain positive in the carcinoma component (brown) and negative in the sarcoma region. **(B)** The CKAE1/3 negative sarcoma (osseous) component at 40x magnification. **(C)** The Type II keratin marker (CK7), **(D)** paired box gene 8 (PAX8) and **(E)** the epithelial membrane antigen (EMA) displays similar staining patterns as CKAE1/3. **(F, G)** The special AT-rich sequence-binding protein 2 (SATB2) marker stains positive for the osteosarcoma component and negative for the carcinoma. **(H, I)** The p53 marker exhibits wild-type expression, with a mixture of negative, weakly positive, and strongly positive cells in both carcinoma and sarcoma components, without signs of mutation, at 40x magnification. IHC staining performed for the mismatch repair proteins, **(J)** MLH1, **(K)** MSH2, **(L)** MSH6 and **(M)** PMS2 showing intact expression in both the carcinoma and sarcoma components. Both components stain negative for **(N)** the cytoskeletal component (Desmin), **(O)** the cell surface marker (CD10) and **(P)** the Wilms’ tumor gene 1 (WT1) marker. Note: IHC was performed in same tissue region (sections with 5µm cuts), to overcome any staining variability.

A detailed battery of immunohistochemical (IHC) studies supported the diagnosis and showed expression of the pancytokeratin (CKAE1/3), CK7, PAX8 and the epithelial membrane antigen (EMA) markers in the carcinoma component, with no immunoreactivity in the sarcoma (osseous) component (**Figures 2A–E**). The sarcoma component stained positive for the SATB2 (**Figures 2F, G**). The p53 immunostaining exhibited a wild-type expression pattern for both components with no evidence of mutation (**Figures 2H, I**), and the IHC for the mismatch repair proteins (MLH1, MSH2, MSH6 and PMS2) displayed an intact staining pattern (**Figures 2J–M**). Additionally, Desmin, CD10 and the WT-1 were negative in both components (**Figures 2N–P**). The tumor staging was Stage1C as per the FIGO classification and stage pT1a, pN0 as per the AJCC guidelines.

The patient tolerated the surgical procedure well and completed 2 out of 4 cycles of paclitaxel and carboplatin adjuvant chemotherapy and brachytherapy. She is currently doing well (based on a 6 month follow up period) and shows no evidence of disease recurrence.

## Discussion

UCS is a rare malignancy, that most commonly affects post-menopausal women of between 60-80 years of age. It has similar risk factors to endometrial adenocarcinoma, including nulliparity, tamoxifen use, estrogen exposure, radiation exposure, tobacco use, alcohol use and obesity (2, 4). Interestingly, the patient we present departs from several of these established commonalities and presents with one of the rarest histological manifestations of this type of cancer, making this case of exceptional interest in comparison with similar reported cases (**Table 1**).

**Table 1:** Summary of limited published articles reporting the occurrence of uterine carcinosarcoma with osteosarcoma (including the current case)^#^.

Paper/Article type/References/Year	Age (years)	Histology	Overall Stage	Treatment	Additional Clinical Information	Survival (at time of publication)
Current CR	31	HG-EC and H-S, (+) O.	Stage IC	TRH/BSO, completed 2 out of 4 cycles of paclitaxel and carboplatin adjuvant CTx and BTx.	No history of malignancy.	Alive, undergoing adjuvant Tx.
Asfer et al./CR/ (26)/2024	74	Malignant epth. carcinoid and H-S, (+) O and (+) C.	Stage II	TAH/BSO, 6 cycles of paclitaxel and carboplatin adjuvant CTx.	(+) pulmonary metastases.	No masses/metastases after adjuvant CTx. Alive, currently under Obs.
Amin et al./CR/ (27)/2023	59	Initial biopsy: ESC Repeat biopsy, 1-year later: ESC and H-S, (+) O.	US	US CTx, radiation and Letrazole.	Patient had past medical history of triple negative breast cancer.	Deceased
Liu Jin et al./CR/ (28)/2018	59	SQ intra-epth metaplasia with EC and H-S, (+) O.	Stage IIIC2	TAH/BSO was performed with bilateral pelvic cavity and periaortic lymphadenectomy, 4 cycles of paclitaxel and carboplatin adjuvant CTx.	1/8^th^ of left side pelvic LNs were (+) for metastases; 1/5^th^ of the right side pelvic LNs were (+) for metastases; two abdominal periaortic LNs (+) metastases.	Alive, no recurrence or metastasis with good quality of life.
Kahraman et al./CR/ (29)/2012	67	EC and H-S, with spindle cells and (+) O.	Stage IVA	TAH/BSO with para-aortic LN dissection. Following surgery, 6 cycles of paclitaxel and carboplatin adjuvant CTx.	Patient History: Sacral chordoma 23 years prior requiring primary surgery. Past medical history: DM and HTN. Evaluation of disease extent: Extensive MM invasion, bilateral paraovarian tissues and the urinary bladder invaded. Ovaries and omentum – no malignancy. Peritoneal fluid cytology (-).	Alive, disease free, 9-months post-operative.
De Brito et al./CS/ (30)/1993	(1): 60 (2):48	(1): HG-EC and H-S, (+) O. (2): UDC and H-S, (+) O.	(1) Stage IC (2) Stage IVB	US	(1): MM and lympho-vascular invasion. (2): Deep MM and lympho-vascular invasion.	(1) 15 months of follow up and alive with disease. (2) Deceased
King et al./CS/ (31)/1980	(1): 66 (2): 78 (3): 61 (4): 71	(1): AC + SQC and H-S, (+) O and (+) R. (2): AC + SQC and H-S, (+) Osteo, (+) R, (+) C. (3): AC and H-S, (+) L, (+) C and (+) O. (4): AC and H-S, (+) L and (+) O.	US	US	(1): Had history of previous radiation; noted to have pleura metastases; tumor invasion unknown. (2): Tumor invasion > 2/3^rd^ of the MM invaded. (3): Tumor invasion maintained within inner 1/3^rd^ of MM. (4): Tumor invasion maintained within inner 1/3^rd^ of MM.	(1): Deceased, 3 months after diagnosis. (2): Deceased, 8 months after diagnosis. (3): Deceased, 15 months after diagnosis. (4): Alive, 5 years after diagnosis.

AC, Adenomatous Carcinoma; BTx, BrachyTherapy; C, Chondrosarcoma; CK, CytoKeratin; CR, Case Report; CS, Case Series; CTx, ChemoTherapy; DM, Diabetes Mellitus; Epth - Epithelial; HG-EC, High Grade Endometrial Carcinoma; H-S, Heterologous Sarcoma; HTN, Hypertension; L, Leiomyosarcoma; LN, Lymph Node; MM, MyoMetrium; (-), Negative; Obs, Observation; O, Osteosarcoma; (+), Positive for; R, Rhabdomyosarcoma; SQC, SQuamous Carcinoma; TRH/TAH/BSO, Total Robotic Hysterectomy/Total Abdominal Hysterectomy/Bilateral Salpingo-Oophorectomy; UDC, UnDifferentiated Carcinoma; US, UnSpecified; V, Vimentin; ^#^Articles published in English language only.

The particularity of this case is its occurrence in a young woman (31-year-old) who is currently responding well to ongoing adjuvant chemotherapy and brachytherapy based on a six month follow up period. During her initial management, several entities were considered in the differential diagnosis. Endometrioid carcinoma with osseous metaplasia was initially considered, however, the presence of malignant features in the osseous component, including high-grade nuclear features, frequent mitoses, and marked cytologic atypia, argued against benign osseous metaplasia. High-grade serous carcinoma was also included in the differential, however, the solid tumor component lacked expression of Müllerian markers such as PAX8, was negative for WT1, and demonstrated wild-type p53 expression, findings that were inconsistent with a diagnosis of high-grade serous carcinoma. Primary osteosarcoma of the uterus was considered but deemed unlikely due to the presence of a high-grade endometrioid carcinoma component, which supports a primary uterine origin. Similarly, dedifferentiated carcinoma was evaluated, as it can exhibit a sarcoma-like morphology. However, the clear separation of the epithelial and sarcomatous components, along with negative epithelial IHC markers in the sarcomatous component argued against this possibility. Finally, metastatic osteosarcoma was also considered but ruled out due to the background endometrioid carcinoma, which strongly supports a primary uterine tumor rather than a metastatic process.

The unique and significant pathological feature of carcinosarcomas is their biphasic nature, with malignant epithelial (carcinomatous) and mesenchymal (sarcomatous) differentiation. The etiology of how this histologic dichotomy comes to manifest in these tumors is still debated, with three suspected mechanisms, namely the collision, combination, and conversion theories (5, 6). The monoclonal origin and epithelial to mesenchymal transition (EMT) of these tumors to a biphasic histologic phenotype is the generally accepted consensus of tumorigenesis substantiated by multiple genetic, molecular and immunohistochemical studies of these tumors (7–10).

The epithelial component the tumor is categorized based on the histologic evaluation, characterized as having either a differentiated endometrioid histology (lower grade) or a poorly differentiated endometrioid, serous or clear cell histology (higher grade) (7, 8, 11). Mutations and/or differential expression of a variety of regulatory genes have been implicated in UCS malignancy and investigated for potential therapeutic exploitation (7, 12). In addition to direct alteration to genetic sequences, loss of heterozygosity and X inactivation are also suggested mechanisms for the propagation of anomalous tumor suppressor protein expression or function (4). While a genetic evaluation was not performed in our patient, having the ability to elucidate the molecular underpinnings of a patient’s cancer allows for a tumor-specific targeted treatment and better prognostic evaluation.

Sarcomatous components are divided into homologous and heterologous subtypes, depending on whether they resemble tissues found naturally in the uterus (4). Homologous sarcomas contain tissues native to the uterus. This includes leiomyosarcoma and endometrial stromal sarcoma. Heterologous sarcomas tend to have elements such as cartilage (chondrosarcoma), skeletal muscle (rhabdomyosarcoma), adipose tissue (liposarcoma), and bone (osteosarcoma). These tissues are not native to the uterus and tumors of this nature are extremely rare. From a mechanistic viewpoint, the sarcomatous cells are believed to undergo EMT in which epithelial cells undergo molecular modification until they achieve a mesenchymal cell type (7).

The current case highlights the importance of comprehensive histopathological and immunohistochemical evaluation, especially in cases of carcinosarcoma with unusual features such as osseous differentiation. In the present case, the carcinomatous component was positive for CKAE1/3, CK7. PAX8 and EMA (**Figures 2A–E**), reliable markers of epithelial cell origin and are therefore specific to the carcinoma portion of the tumor (13). In contrast, the sarcomatous component was negative for CK7 and CKAE 1/3 and positive for SATB2, consistently associated with tissues having osseous elements (**Figures 2F, G**). SATB2, while highly sensitive for osseous components, may lack specificity (14, 15). Therefore, while SATB2 is useful in ruling out osseous pathology additional measures should be implemented to confirm diagnosis.

Molecular drivers seen in more common presentations of endometrial cancers have also been investigated to see if there is mutual association with carcinosarcoma. Specifically, mutations in mismatch repair genes (MLH1, MSH2, MSH6, and PMS2) associated with Lynch syndrome (16–18). Mismatch repair proteins and the relative expression of PD-L1 have also been studied for potential utility of PD-L1 inhibitors in these cancers (19, 20). The immunohistochemical evaluation of the resected tissue in our case revealed no association with Lynch syndrome (intact expression of the 4 mismatch repair proteins, **Figures 2J–M**). Overall, the association between mismatch repair protein defects and carcinosarcoma appears infrequent and may be of limited utility.

In addition to the canonical prognostic elements (tumor size, depth of tissue invasion, metastases, etc.) both the carcinomatous and sarcomatous components contribute to the biological behavior of carcinosarcoma and affect survival (4). The carcinomatous elements of the tumor have been found to direct tumor progression and metastases (4, 8). The rare heterologous components, such as osteosarcoma in the present case, complicate both diagnosis and treatment. Studies are mixed regarding the significance of heterologous elements and their effect on patient prognosis (4, 8, 21). A recent meta-analysis by Kim et al. evaluated eight studies, including 1,594 patients, which evaluated survival in patients with uterine and ovarian carcinosarcomas (22). Their study found a significantly worse overall survival with heterologous sarcoma, but this effect was not maintained in a pooled analysis of recurrence free survival or disease-free survival. Interestingly, the percentage of tumor morphology tends to significantly affect prognosis. Specifically, tumors which are predominately (>50%) comprised of sarcomatous elements, regardless of type (heterologous or homologous), tend to have decreased survival (4, 23). Finally, it is noteworthy that UCS, have a high rate of metastasis, with carcinomatous tissue grade determining the malignant potential. With these factors in mind, accurate and timely histologic evaluation of these tumors is of the utmost importance.

Despite the rarity of osteosarcoma as a heterologous component, it is crucial to identify these elements because they may adversely influence the tumor’s biological behavior and response to treatment. Current National Comprehensive Cancer Network (NCCN) guidelines for management of carcinosarcoma include surgical resection, if the patient is deemed able to tolerate surgery, including a total abdominal hysterectomy with bilateral salpingoophorectomy with tumor debulking and staging (24). In the treatment of carcinosarcoma, chemotherapy regimens with the highest efficacy primarily consist of a combination of a taxane and platinum-based chemotherapeutic or ifosfamide (3, 4, 25). Per the NCCN guidelines for management of uterine carcinosarcoma, regardless of stage, adjuvant systemic chemotherapy is recommended and vaginal brachytherapy interspersed between cycles of chemotherapy is conditionally recommended (24). External beam radiation therapy is conditionally recommended as well, specifically for cases with high-grade carcinoma or those with sarcoma dominance (25). As therapeutic approaches for uterine carcinosarcoma continue to evolve, including the use of adjuvant chemotherapy and radiotherapy, accurate characterization of both the epithelial and sarcomatous components remains vital for guiding prognosis and treatment strategies.

## Conclusion

UCS with osteosarcomatous heterologous elements is an exceedingly rare and aggressive tumor. This case highlights the importance of considering UCS in younger patients, as the patient in this case was 31 years old. Early recognition and diagnosis in this age group can significantly impact management and outcomes, underscoring the need for heightened awareness among clinicians when encountering similar presentations in younger populations. The biphasic nature of these tumors necessitates detailed pathological assessment to identify both components and any heterologous differentiation, which may significantly impact prognosis and therapeutic decisions. Further studies with larger sample sizes are needed to better understand the behavior of these rare variants and to optimize treatment protocols.

## Data Availability

The original contributions presented in the study are included in the article/supplementary material. Further inquiries can be directed to the corresponding author.

## References

[1] Nama N Cason FD Misra S Hai S Tucci V Haq F. Carcinosarcoma of the uterus: A study from the surveillance epidemiology and end result (Seer) database. Cureus. (2020) 12:e10283. doi: 10.7759/cureus.10283 33042718 PMC7538206

[2] Tang LS Zhou YW Wang JL Zhang GX Xu CH Liu JY. Epidemiology, site-specific characteristics and survival of carcinosarcoma: A retrospective study based on seer database. BMJ Open. (2023) 13:e077974. doi: 10.1136/bmjopen-2023-077974 PMC10729011 38101828

[3] Cantrell LA Blank SV Duska LR. Uterine carcinosarcoma: A review of the literature. Gynecol Oncol. (2015) 137:581–8. doi: 10.1016/j.ygyno.2015.03.041 25805398

[4] Kernochan LE Garcia RL. Carcinosarcomas (Malignant mixed müllerian tumor) of the uterus: advances in elucidation of biologic and clinical characteristics. J Natl Compr Canc Netw. (2009) 7:550–556; quiz 557. doi: 10.6004/jnccn.2009.0037 19460280

[5] Somarelli JA Boss MK Epstein JI Armstrong AJ Garcia-Blanco MA. Carcinosarcomas: tumors in transition? Histol Histopathol. (2015) 30:673–87. doi: 10.14670/hh-30.673 25587806

[6] Pang A Carbini M Moreira AL Maki RG. Carcinosarcomas and related cancers: tumors caught in the act of epithelial-mesenchymal transition. J Clin Oncol. (2018) 36:210–6. doi: 10.1200/jco.2017.74.9523 29220296

[7] Leskela S Pérez-Mies B Rosa-Rosa JM Cristobal E Biscuola M Palacios-Berraquero ML. Molecular basis of tumor heterogeneity in endometrial carcinosarcoma. Cancers. (2019) 11:964. doi: 10.3390/cancers11070964 31324031 PMC6678708

[8] Matsuzaki S Klar M Matsuzaki S Roman LD Sood AK Matsuo K. Uterine carcinosarcoma: contemporary clinical summary, molecular updates, and future research opportunity. Gynecol Oncol. (2021) 160:586–601. doi: 10.1016/j.ygyno.2020.10.043 33183764

[9] de Jong RA Nijman HW Wijbrandi TF Reyners AK Boezen HM Hollema H. Molecular markers and clinical behavior of uterine carcinosarcomas: focus on the epithelial tumor component. Mod Pathol. (2011) 24:1368–79. doi: 10.1038/modpathol.2011.88 21572397

[10] Chen X Arend R Hamele-Bena D Tergas AI Hawver M Tong G-X. Uterine carcinosarcomas: clinical, histopathologic and immunohistochemical characteristics. Int J Gynecological Pathol. (2017) 36:412–9. doi: 10.1097/pgp.0000000000000346 28700424

[11] Cherniack AD Shen H Walter V Stewart C Murray BA Bowlby R. Integrated molecular characterization of uterine carcinosarcoma. Cancer Cell. (2017) 31:411–23. doi: 10.1016/j.ccell.2017.02.010 PMC5599133 28292439

[12] Hanley KZ Horowitz IR Gordon A Meisel J Khanna N. Folate receptor alpha is preferentially expressed in the carcinoma component of endometrial carcinosarcomas: A potential target for adjuvant therapy. Int J Gynecological Pathol. (2021) 40:501–9. doi: 10.1097/pgp.0000000000000736 33323854

[13] Kandukuri SR Lin F Gui L Gong Y Fan F Chen L. Application of immunohistochemistry in undifferentiated neoplasms: A practical approach. Arch Pathol Lab Med. (2017) 141:1014–32. doi: 10.5858/arpa.2016-0518-RA 28745568

[14] Sangoi AR Kshirsagar M Horvai AE Roma AA. Satb2 expression is sensitive but not specific for osteosarcomatous components of gynecologic tract carcinosarcomas: A clinicopathologic study of 60 cases. Int J Gynecol Pathol. (2017) 36:140–5. doi: 10.1097/pgp.0000000000000301 27294605

[15] Davis JL Horvai AE. Special at-Rich Sequence-Binding Protein 2 (Satb2) Expression Is Sensitive but May Not Be Specific for Osteosarcoma as Compared with Other High-Grade Primary Bone Sarcomas. Histopathology. (2016) 69:84–90. doi: 10.1111/his.12911 26644288

[16] Hoang LN Ali RH Lau S Gilks CB Lee C-H. Immunohistochemical survey of mismatch repair protein expression in uterine sarcomas and carcinosarcomas. Int J Gynecological Pathol. (2014) 33:483–91. doi: 10.1097/PGP.0b013e31829ff239 25083964

[17] Jenkins TM Hanley KZ Schwartz LE Cantrell LA Stoler MH Mills AM. Mismatch repair deficiency in uterine carcinosarcoma: A multi-institution retrospective review. Am J Surg Pathol. (2020) 44:782–92. doi: 10.1097/pas.0000000000001434 31934920

[18] Taylor NP Zighelboim I Huettner PC Powell MA Gibb RK Rader JS. DNA mismatch repair and tp53 defects are early events in uterine carcinosarcoma tumorigenesis. Modern Pathol. (2006) 19:1333–8. doi: 10.1038/modpathol.3800654 16810312

[19] Jones TE Pradhan D Dabbs DJ Bhargava R Onisko A Jones MW. Immunohistochemical markers with potential diagnostic, prognostic, and therapeutic significance in uterine carcinosarcoma: A clinicopathologic study of 43 cases. Int J Gynecological Pathol. (2021) 40:84–93. doi: 10.1097/pgp.0000000000000662 31855950

[20] Jenkins TM Cantrell LA Stoler MH Mills AM. Pd-L1 and mismatch repair status in uterine carcinosarcomas. Int J Gynecological Pathol. (2021) 40:563–74. doi: 10.1097/pgp.0000000000000752 33323859

[21] Matsuo K Takazawa Y Ross MS Elishaev E Podzielinski I Yunokawa M. Significance of histologic pattern of carcinoma and sarcoma components on survival outcomes of uterine carcinosarcoma. Ann Oncol. (2016) 27:1257–66. doi: 10.1093/annonc/mdw161 27052653

[22] Kim Y Kang GH Kim H. Prognostic significance of heterologous component in carcinosarcoma of the gynecologic organs: A systematic review and meta-analysis. J Gynecol Oncol. (2023) 34:e73. doi: 10.3802/jgo.2023.34.e73 37417301 PMC10627759

[23] Matsuo K Takazawa Y Ross MS Elishaev E Yunokawa M Sheridan TB. Characterizing sarcoma dominance pattern in uterine carcinosarcoma: homologous versus heterologous element. Surg Oncol. (2018) 27:433–40. doi: 10.1016/j.suronc.2018.05.017 PMC7526039 30217299

[24] Abu-Rustum N Yashar C Arend R Barber E Bradley K Brooks R. Uterine Neoplasms, Version 3 (2024). National Comprehensive Cancer Network - Clinical Practice Guidelines in Oncology (Nccn Guidelines (Accessed September 26th, 2024).

[25] Matsuo K Omatsu K Ross MS Johnson MS Yunokawa M Klobocista MM. Impact of adjuvant therapy on recurrence patterns in stage I uterine carcinosarcoma. Gynecologic Oncol. (2017) 145:78–87. doi: 10.1016/j.ygyno.2017.02.001 PMC7529360 28215838

[26] Asfer S Hmidan Samsam S Zakkar R Jarbouh H. Uterine carcinosarcoma with heterologous mesenchymal element: A case report of a rare and aggressive tumor. Oxf Med Case Rep. (2024) 2024:omad157. doi: 10.1093/omcr/omad157 PMC10873701 38370501

[27] Amin SE Elzamly S Ahmed AA Tandon N. Uterine carcinosarcoma presenting as metastatic osteosarcoma in the lung: A case report and literature review. Ann Clin Lab Sci. (2023) 53:969–73. 38182153

[28] Jin L Xi Y Wenyuan Z Jinliang P Liping Z Shuwen H. Osteosarcoma appearing in the uterus as Malignant mixed müllerian tumor. Eur J Gynaecological Oncol. (2018) 39:488–92. doi: 10.12892/ejgo3991.2018

[29] Kahraman K Ortac F Kankaya D Aynaoglu G. Uterine carcinosarcoma associated with pelvic radiotherapy for sacral chordoma: A case report. Taiwanese J Obstetrics Gynecology. (2012) 51:89–92. doi: 10.1016/j.tjog.2012.01.018 22482976

[30] de Brito PA Silverberg SG Orenstein JM. Carcinosarcoma (Malignant mixed müllerian (Mesodermal) tumor) of the female genital tract: immunohistochemical and ultrastructural analysis of 28 cases. Hum Pathol. (1993) 24:132–42. doi: 10.1016/0046-8177(93)90291-N 7679366

[31] King ME Kramer EE. Malignant mullerian mixed tumors of the uterus a study of 21 cases. Cancer. (1980) 45:188–90. doi: 10.1002/1097-0142(19800101)45:1<188::AID-CNCR2820450129>3.0.CO;2-Y 6243241

